# Seven new species of jumping spiders (Araneae, Salticidae) from Xishuangbanna, China

**DOI:** 10.3897/zookeys.968.55047

**Published:** 2020-09-16

**Authors:** Cheng Wang, Shuqiang Li

**Affiliations:** 1 College of Agriculture and Forestry Engineering and Planning, Tongren University, Tongren, Guizhou, 554300, China Tongren University Tongren China; 2 Institute of Zoology, Chinese Academy of Sciences, Beijing 100101, China Chinese Academy of Sciences Beijing China

**Keywords:** distribution, genus, salticid, taxonomy, Yunnan

## Abstract

Seven new species of jumping spiders collected from Xishuangbanna Tropical Botanical Garden in Yunnan, China, are diagnosed and described: *Charippus yinae***sp. nov.** (♂♀), *Chinattus inflatus***sp. nov.** (♂), *Indomarengo yui***sp. nov.** (♂), *Phintella banna***sp. nov.** (♂♀), *P. mii***sp. nov.** (♂♀), *Simaetha menglun***sp. nov.** (♂♀) and *S. pengi***sp. nov.** (♂♀). *Charippus yinae***sp. nov.** is the second species of the genus *Charippus* Thorell, 1895, which was previously known only from one sex.

## Introduction

Xishuangbanna is a key biogeographic area and a biodiversity hotspot in China (Myers 1988). Spider diversity in the area is high, with 782 species spanning 305 genera in 46 families (Li 2020). The Salticidae Blackwall, 1841 from this region have been studied by Peng and Yin (1991), Song (1991), Peng (1995), Xie and Peng (1995), Peng and Kim (1997), Song and Zhu (1998), Xiao (2002), Xiao and Wang (2004), Cao et al. (2016), Wang and Li (2020), and Lin and Li (2020). These studies have resulted in the description of 45 new species and increased the total salticid species number to 121 in the last 30 years (Lin and Li 2020). At least 19 salticid species described as new from Xishuangbanna are known only from a single sex, and sparsely collected new species are frequently being discovered as a result of the ongoing project “All Species Inventory” of spiders from the Xishuangbanna Tropical Botanical Garden. Our results thus far indicate that the salticid fauna of Xishuangbanna remains insufficiently known and requires further research.

A taxonomic study on the recently available salticid samples of Xishuangbanna has revealed seven species that are new to science and are described here. These results increase the total number of salticid species in the region to 128.

## Materials and methods

Specimens were collected by fogging and sieving leaf litter in the tropical rainforest of Xishuangbanna Tropical Botanical Garden, Yunnan, China. All specimens were preserved in 75% ethanol and are deposited in the Institute of Zoology, Chinese Academy of Sciences (**IZCAS**) in Beijing, China. The specimens were examined with an Olympus SZ51 stereomicroscope. After dissection, the epigyne was cleared in trypsin enzyme solution before examination and imaging. Images of the copulatory organs and habitus were taken with a Kuy Nice CCD mounted on an Olympus BX51 compound microscope. Compound focus images were generated using Helicon Focus v. 6.7.1.

All measurements are given in millimeters. Leg measurements are given as: total length (femur, patella + tibia, metatarsus, tarsus). References to figures in the cited papers are listed in lowercase type (fig. or figs); figures in this paper are noted with an initial capital (Fig. or Figs). Abbreviations used in the text and figures are as follows:

**AAM** anterior atrial margin;

**AER** anterior eye row;

**AERW** anterior eye row width;

**ALE** anterior lateral eye;

**AME** anterior median eye;

**AS** anterior chamber of spermatheca;

**BP** basal plate;

**CD** copulatory duct;

**CO** copulatory opening;

**CP** cymbial process;

**E** embolus;

**EC** embolic coil;

**EFL** eye field length;

**F** fold;

**FD** fertilization duct;

**H** hood;

**LAM** lateral atrial margin;

**LP** lamellar process;

**PERW** posterior eye row width;

**PL** posterior lobe;

**PLE** posterior lateral eye;

**PS** posterior chamber of spermatheca;

**RFA** retrolateral femoral apophysis;

**RTA** retrolateral tibial apophysis;

**S** spermatheca;

**SD** sperm duct;

**TB** tegular bump.

## Taxonomy

### Family Salticidae Blackwall, 1841

#### Genus Charippus

Taxon classificationAnimaliaAraneaeSalticidae

Thorell, 1895

E54410E6-FABE-5A86-8932-9927A95AC2DC

##### Type species.

*Charippus errans* Thorell, 1895

##### Comments.

This monotypic genus is only known from the descriptions of the generotype male (Thorell 1895; WSC 2020). *Charippus* is similar to *Cytaea* Keyserling, 1882 by the general shape of the copulatory organs, especially the spiral embolus and the long and coiled copulatory ducts but can be distinguished by the following: 1) the carapace is slightly narrowed anteriorly vs almost square in *Cytaea* species; 2) the color of the habitus is dark rather than light, as in *Cytaea* species, indicating that *Charippus* likely comprises leaf-litter dwellers rather than foliage dwellers, as in *Cytaea*; 3) the RTA is distinctly longer than in *Cytaea* species; 4) the male chelicerae only have two promarginal teeth vs at least three promarginal teeth in *Cytaea* species; 5) the epigyne lacks a distinct median septum, which is present in *Cytaea* species.

#### Charippus yinae sp. nov.

Taxon classificationAnimaliaAraneaeSalticidae

00F9EBBD-7759-5A93-8837-0A255A7A68C3

http://zoobank.org/97A61C13-E1F2-49D7-8074-0404C15CDD05

Figs 1 , 2

##### Type material.

***Holotype*** ♂ (IZCAS-Ar40601), China: Yunnan: Xishuangbanna, Mengla County, Menglun Town, Menglun Nature Reserve, Xishuangbanna Tropical Botanical Garden, Leprosy Village (21°53.59'N, 101°17.30'E, ca 550 m), 29.04.2019, Y. Tong et al. leg. ***Paratypes*** 1♀ (IZCAS-Ar40602), same data as holotype; 1♀ (IZCAS-Ar40603), Flower Garden (21°55.80'N, 101°45.41'E, ca 530 m), 1.08.2018, C. Wang et al. leg.

##### Etymology.

The species name is a patronym in honor of the late Professor Changmin Yin, one of the pioneers of spider taxonomy of China; noun (name) in genitive case.

##### Diagnosis.

The male of *Charippus yinae* sp. nov. resembles *C. errans* Thorell, 1895 by the habitus, shape of the palp, and the cheliceral dentition, but can be easily distinguished by the embolus originating at 9:00 o’clock rather than approximately 11:30 o’clock as in *C. errans* (Wanless 1988: fig. 40F), and the RTA is curved towards the bulb medially in retrolateral view, instead of curved towards the bulb terminally as in *C. errans* (Wanless 1988: fig. 40H). The female resembles *Cytaea alburna* Keyserling, 1882 by the very long, coiled copulatory ducts and the small spermathecae but can be easily distinguished by the following: 1) the carapace is slightly narrowed anteriorly rather than almost square as in *C. alburna* (Davies and Żabka 1989: 226); 2) the median septum is absent vs present in *C. alburna* (Davies and Żabka 1989: 226); 3) the chelicerae have only two promarginal teeth vs three promarginal teeth as in *C. alburna* (Davies and Żabka 1989: 226).

##### Description.

**Figure 1.**
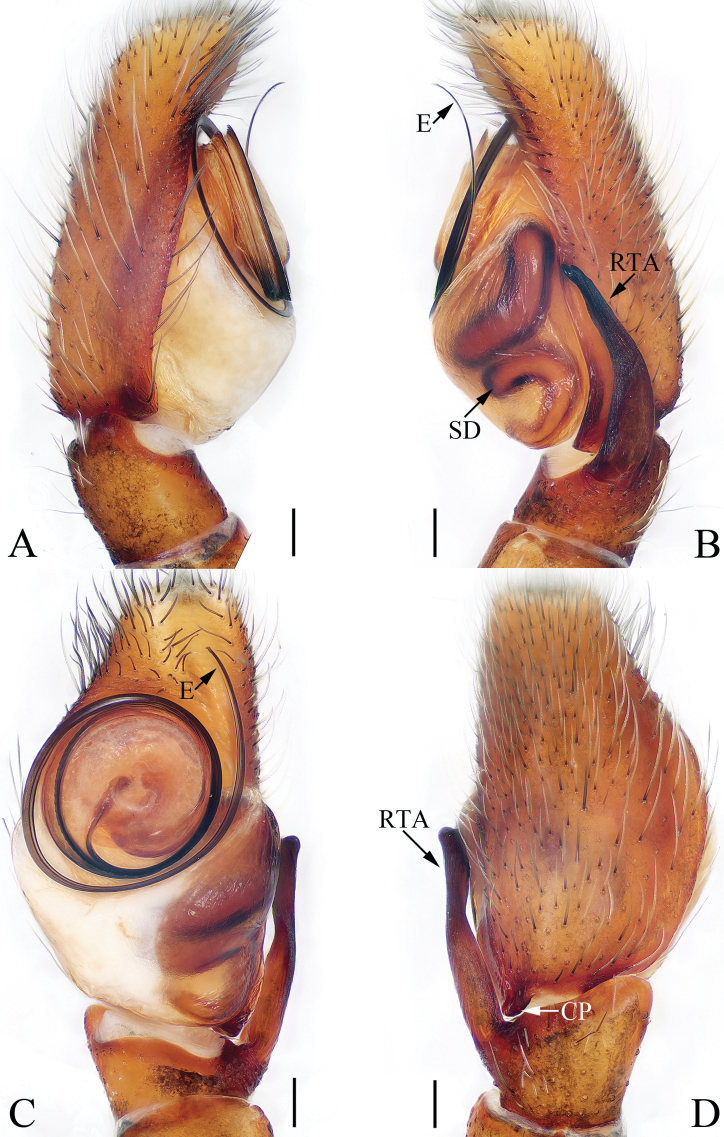
Male palp of the holotype of *Charippus yinae* sp. nov. **A** prolateral **B** retrolateral **C** ventral **D** dorsal. Scale bars: 0.1.

**Figure 2.**
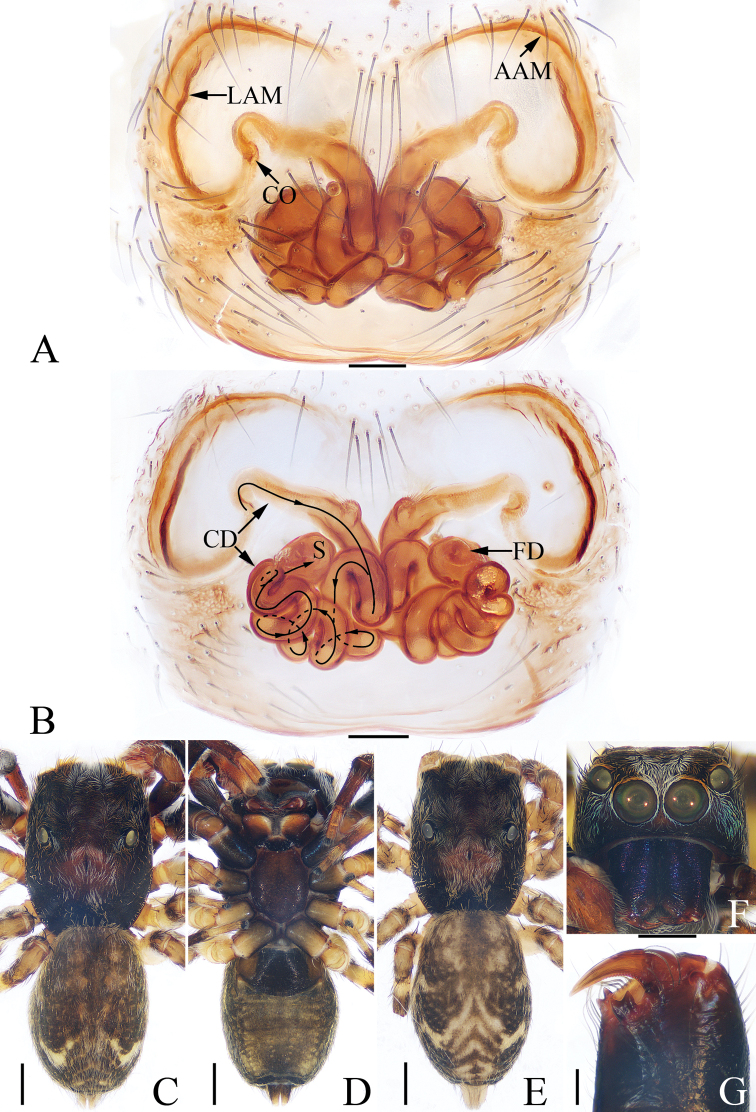
*Charippus yinae* sp. nov., female paratype and male holotype **A** epigyne, ventral **B** vulva, dorsal **C** holotype habitus, dorsal **D** holotype habitus, ventral **E** paratype habitus, dorsal **F** holotype carapace, frontal **G** holotype chelicerae, dorsal. Scale bars: 0.1 (**A, B, G**); 0.5 (**C–F**).

**Male** (Figs 1, 2C, D, F, G). Total length 3.91. Carapace 1.98 long, 1.56 wide. Abdomen 2.04 long, 1.48 wide. Clypeus 0.10 high. Eye sizes and inter-distances: AME 0.38, ALE 0.22, PLE 0.20, AERW 1.28, PERW 1.24, EFL 0.97. Legs: I 3.83 (1.15, 1.61, 0.68, 0.39), II 3.53 (1.07, 1.39, 0.68, 0.39), III 3.78 (1.22, 1.29, 0.88, 0.39), IV 4.27 (1.22, 1.49, 1.07, 0.49). Carapace dark brown, with an irregular red area in the middle of the thoracic part, bearing dense white setae on the cheeks and white and golden setae dorsally. Clypeus dark, with sparse white setae. Fovea longitudinal, dark. Chelicerae red to dark brown, with two promarginal teeth and one retromarginal fissident with two cusps. Endites red-brown, paler inner margin. Labium dark brown. Sternum somewhat shield-shaped, with sparse setae. Legs I red to dark brown; other legs yellow, with dark markings. Abdomen suboval, dorsum hirsute, with two pairs of faint pale patches anteriorly and a pair of posterolateral falcate bands followed by several chevrons; venter dark anteriorly, covered by small spots and thin brown setae.

***Palp*** (Fig. 1A–D): tibia wider than long in ventral view, with a RTA approximately two times the tibial length, slightly curved medially and blunt at the tip in retrolateral view; cymbium hirsute, proximodorsally with a small process (Fig. 1D, CP) near the RTA base; bulb swollen, with sperm duct strongly curved retrolaterally; embolus originating at around 9:00 o’clock, coiled 1.5 times.

**Female** (Fig. 2A, B, E). Total length 3.83. Carapace 1.83 long, 1.38 wide. Abdomen 2.13 long, 1.45 wide. Clypeus 0.10 high. Eye sizes and inter-distances: AME 0.36, ALE 0.23, PLE 0.21, AERW 1.20, PERW 1.14, EFL 0.88. Legs: I 3.08 (0.98, 1.27, 0.49, 0.34), II 2.88 (0.95, 1.10, 0.49, 0.34), III 3.32 (1.10, 1.15, 0.73, 0.34), IV 3.58 (1.12, 1.29, 0.83, 0.34). Habitus similar to that of male except slightly paler.

***Epigyne*** (Fig. 2A, B): wider than long, with a pair of arc-shaped anterior atrial margins and a pair of lateral atrial margins; copulatory openings small, slit-shaped, located anterolaterally; copulatory ducts very long, curved into a U-shape at the origin, with anterior tubercles and complex coils; spermathecae suboval, separated from each other by almost two times their width; fertilization ducts nearly cordiform.

##### Distribution.

Known only from the type locality in Yunnan, China.

#### Genus Chinattus

Taxon classificationAnimaliaAraneaeSalticidae

Simon, 1876

CFA18007-E7EA-531F-B2D1-A17D5DC2D017

##### Type species.

*Habrocestoides szechwanensis* Prószyński, 1992

##### Comments.

The genus is represented by 17 nominal species restricted to Asia, except *C. parvulus* (Banks, 1895), which is known from the USA and Canada. Diagnostic drawings have been made for all species in the genus, and nearly all have limited distributional ranges: 11 species are endemic, and five species are known only from two countries. Six species are known only from a single sex: two from males and four from females (all from China). To date, 12 species are recorded from China. Of those, eight are endemic (WSC 2020).

#### Chinattus inflatus sp. nov.

Taxon classificationAnimaliaAraneaeSalticidae

485CC0C5-CFD3-576D-9FF0-57B8053ADBE0

http://zoobank.org/6BD4F191-4595-492A-8794-6480493017BB

Figs 3 , 4

##### Type material.

***Holotype*** ♂ (IZCAS-Ar40604), China: Yunnan: Xishuangbanna, Mengla County, Menglun Town, Menglun Nature Reserve, Xishuangbanna Tropical Botanical Garden, tropical rainforest (21°55.20'N, 101°16.21'E, ca 550 m), 30.04.2019, Y. Tong et al. leg. ***Paratype*** 1♂ (IZCAS-Ar40605), same data as holotype.

##### Etymology.

The specific name refers to the inflated femur of the male palp; adjective.

##### Diagnosis.

*Chinattus inflatus* sp. nov. resembles *C. wengnanensis* Cao & Li, 2016 in the general shape of the habitus and male palp but can be easily distinguished by the inflated palpal femur, the femoral apophysis and the long embolus. The new species also resembles species of the genus *Grayenulla* Żabka, 1992 by the inflated palpal femur and the femoral apophysis but can be distinguished by leg III, which is one of the shortest legs rather than distinctly the longest as in species of *Grayenulla*. Additionally, the clypeus lacks bristles, whereas there are three central bristles in species of *Grayenulla* (Żabka 1992).

##### Description.

**Figure 3.**
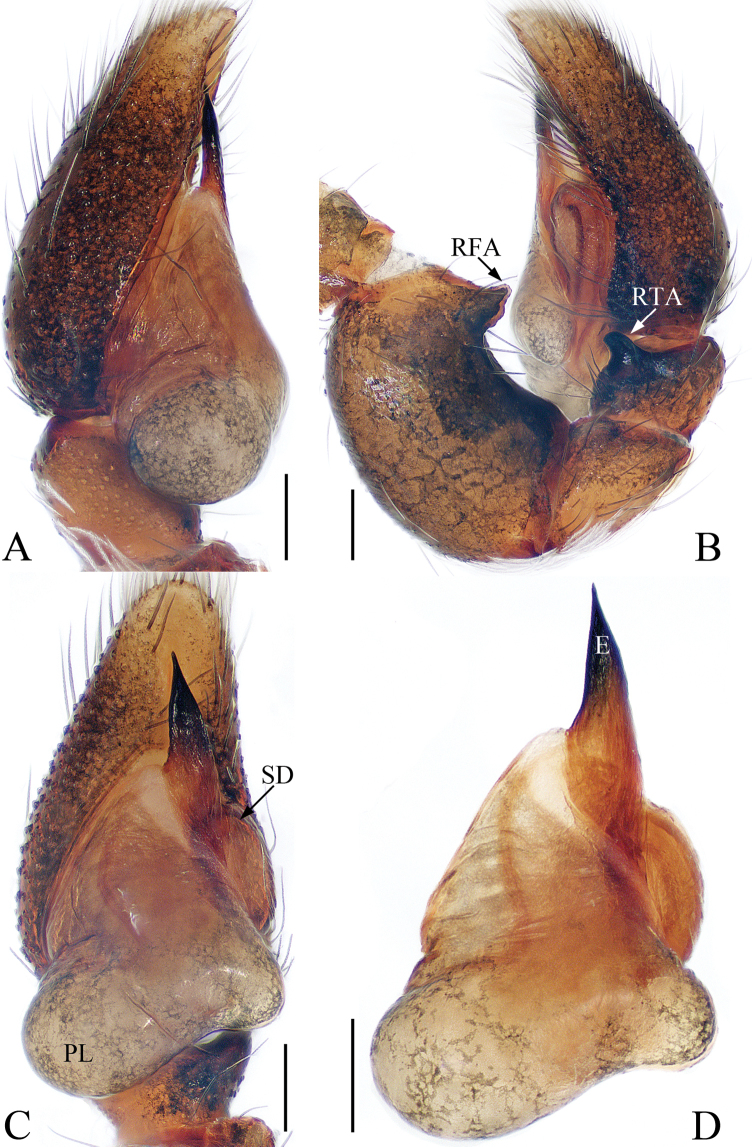
Male palp of the holotype of *Chinattus inflatus* sp. nov. **A** prolateral **B** retrolateral **C** ventral **D** bulb, ventral. Scale bars: 0.1.

**Figure 4.**
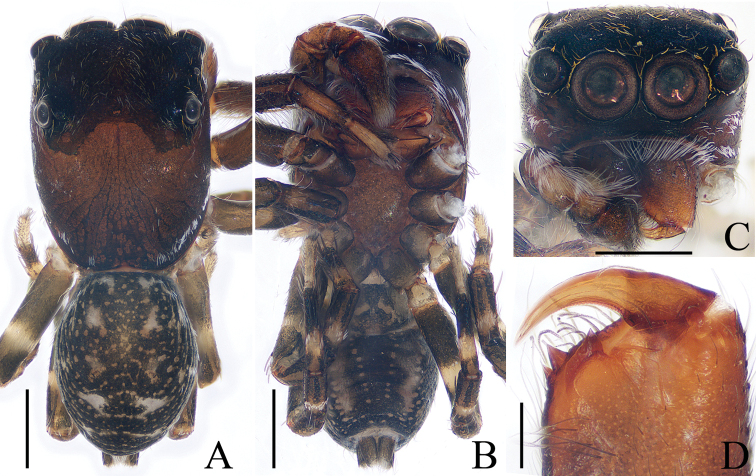
*Chinattus inflatus* sp. nov., male holotype **A** habitus, dorsal **B** habitus, ventral **C** carapace, frontal **D** chelicerae, dorsal. Scale bars: 0.5 (**A–C**); 0.1 (**D**).

**Male** (Figs 3, 4). Total length 2.72. Carapace 1.53 long, 1.14 wide. Abdomen 1.19 long, 0.92 wide. Clypeus 0.10 high. Eye sizes and inter-distances: AME 0.36, ALE 0.21, PLE 0.17, AERW 1.13, PERW 1.08, EFL 0.57. Legs: I 2.82 (0.85, 1.12, 0.46, 0.39), II 2.38 (0.73, 0.85, 0.41, 0.39), III 2.65 (0.85, 0.85, 0.56, 0.39), IV 2.75 (0.88, 0.85, 0.63, 0.39). Carapace brown, dark in eye field, bearing white, thin setae on the lateral submargin, and golden, thin setae on the cheeks and cephalic region. Clypeus dark. Fovea longitudinal, short, bar-shaped. Chelicerae red-brown, with two promarginal teeth and one retromarginal fissident. Endites, labium and sternum paler than chelicerae. Legs red-brown to dark brown, with pale rings on femora and metatarsi. Abdomen suboval, dorsum brown, speckled, with three pairs of irregular white spots and two pairs of muscle depressions; venter darker than dorsum, with a pair of off-white longitudinal bands laterally and a pair of dotted lines medially.

***Palp*** (Fig. 3A–D): femur inflated, more than 1.5 times longer than wide, with a subtriangular retrolateral apophysis proximally; tibia wider than long, with a sclerotized, short RTA extending towards the bulb in retrolateral view and almost completely hidden by the bulb in ventral view; posterior lobe well-developed, with oval margin; embolus flat and straight, tapered to a pointed tip.

**Female.** Unknown.

##### Distribution.

Known only from the type locality in Yunnan, China.

##### Comments.

The species is placed into this genus because it generally resembles *C. wengnanensis* Cao & Li, 2016. It is described only based on males, and so there is a possibility that it is conspecific to one of the congeners known only from females.

#### Genus Indomarengo

Taxon classificationAnimaliaAraneaeSalticidae

Benjamin, 2004

30B6329B-3447-542F-B63B-8896F435B453

##### Type species.

*Indomarengo sarawakensis* Benjamin, 2004

##### Comments.

This genus only contains four species, all endemic: one from Borneo, two from Indonesia and one from India. Two are known only from a single sex: *I. chandra* Benjamin, 2004 from males and *I. thomsoni* (Wanless 1978) from females (WSC 2020). The genus can be distinguished from all other Ballini genera, except *Sadies* Wanless, 1984, *Leikung* Benjamin, 2004, and *Afromarengo* Benjamin, 2004, by the presence of a carapace protuberance. It can be distinguished from *Afromarengo* and *Leikung* by the short embolus, which has less than two spirals, and from *Sadies* by the lack of leaf-like carapace scales (Benjamin 2004).

#### Indomarengo yui sp. nov.

Taxon classificationAnimaliaAraneaeSalticidae

02093CE8-C595-5185-9D26-EC11412F3BD4

http://zoobank.org/6CA2DCC9-71E9-488B-9A3F-29B3E59E09AC

Figs 5 , 6

##### Type material.

***Holotype*** ♂ (IZCAS-Ar40606), China: Yunnan: Xishuangbanna, Mengla County, Menglun Town, Menglun Nature Reserve, garbage dump, secondary tropical rainforest (21°54.30'N, 101°16.78'E, ca 620 m), 26.04.2019, H. Yu et al. leg. ***Paratype*** 1♂ (IZCAS-Ar40607), same data as holotype.

##### Etymology.

The specific name is a patronym after Prof. Hao Yu (Guiyang, China), one of the collectors of the new species; noun (name) in genitive case.

##### Diagnosis.

*Indomarengo yui* sp. nov. resembles *I. chavarapater* Malamel, Prajapati, Sudhikumar & Sebastian, 2019 by the general shape of the palp but can be distinguished by the RTA, which is distally curved towards the bulb in retrolateral view vs almost straight and directed anteriorly in *I. chavarapater* (Malamel et al. 2019: figs 11, 15); and by the tibia, which is wider than long vs almost as long as wide in *I. chavarapater* (Malamel et al. 2019: figs 10, 11, 15, 16).

**Figure 5.**
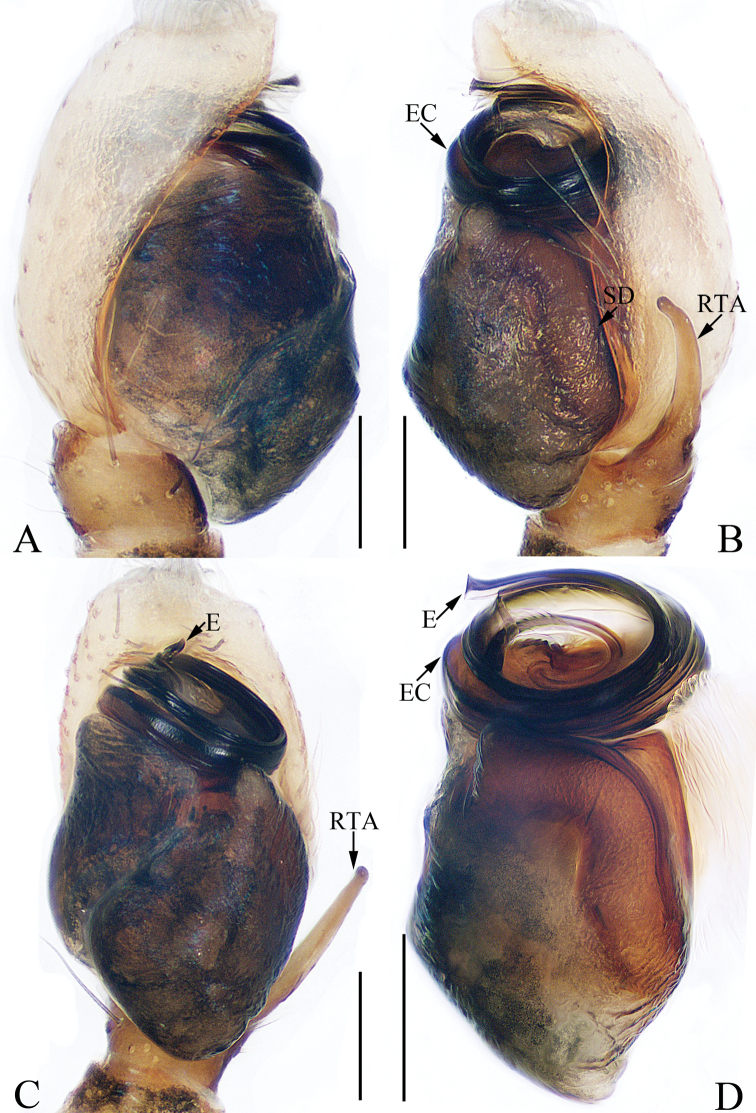
Male palp of the holotype of *Indomarengo yui* sp. nov. **A** prolateral **B** retrolateral **C** ventral **D** bulb, retrolateral. Scale bars: 0.1.

##### Description.

**Figure 6.**
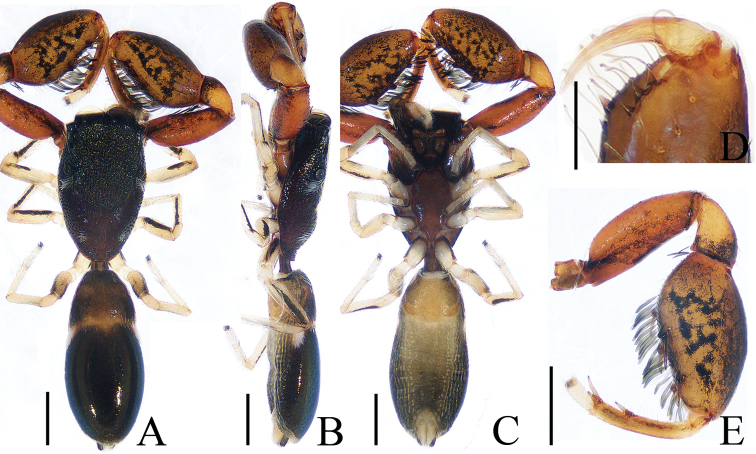
*Indomarengo yui* sp. nov., male holotype **A** habitus, dorsal **B** habitus, lateral **C** habitus, ventral **D** chelicerae, dorsal **E** left leg I, prolateral. Scale bars: 0.5 (**A–C, E**); 0.1 (**D**).

**Male** (Figs 5, 6). Total length 3.12. Carapace 1.36 long, 0.85 wide. Abdomen 1.67 long, 0.76 wide. Clypeus 0.05 high. Eye sizes and inter-distances: AME 0.29, ALE 0.12, PLE 0.12, AERW 0.75, PERW 0.78, EFL 0.53. Legs: I 3.46 (0.93, 1.51, 0.80, 0.22), II 1.79 (0.54, 0.66, 0.37, 0.22), III 1.67 (0.49, 0.59, 0.37, 0.22), IV 2.09 (0.63, 0.78, 0.46, 0.22). Carapace flat, red-brown to dark brown, there are white setae behind the PLEs and posteriorly, with a distinct protuberance on the thoracic region. Clypeus dark. Fovea indistinct. Chelicerae red-brown, with three retromarginal teeth and two promarginal teeth. Endites and labium dark brown. Sternum red-brown, elongated and tapered posteriorly, covered with thin setae. Legs I enlarged, with inflated tibia bearing ventral leaf-shaped scales and five spines; other legs pale, with dark stripes laterally on femora and patellae. Abdomen elongated oval, dorsum brown to dark brown, constricted at first anterior third, with white setae at lateral margins; venter pale to brown.

***Palp*** (Fig. 5A–D): tibia wider than long, with a thin RTA approximately two times the tibial length, widened at the base and curved towards bulb terminally; bulb inflated, divided by a furrow; embolus short, coiled, forming two circles, and partly covered by a membranous structure.

**Female.** Unknown.

##### Distribution.

Known only from the type locality in Yunnan, China.

##### Comments.

The species has a distinct carapace protuberance, which is only known to occur in species of the genera *Indomarengo* Benjamin, 2004, *Sadies* Wanless, 1984, *Leikung* Benjamin, 2004, and *Afromarengo* Benjamin, 2004 of the Ballini. Based on similarities such as the short embolus with less than two spirals and lack of leaf-like carapace scales (more than two spirals in *Leikung* and *Afromarengo* species and leaf-like carapace scales present in *Sadies* species) in addition to closely resembling *I. chavarapater* Malamel, Prajapati, Sudhikumar & Sebastian, 2019 by the habitus and the shape of the male palp, we place the spider into this genus.

#### Genus Phintella

Taxon classificationAnimaliaAraneaeSalticidae

Strand, 1906

61C1212C-5072-560E-8F53-179810E61C97

##### Type species.

*Telamonia bifurcilinea* Bösenberg & Strand, 1906

##### Comments.

*Phintella* is a rather large genus, represented by a group of small and colorful spiders, typically covered with metallic iridescent scales (Luong et al. 2016). A total of 60 nominal species have been described, primarily from the Afrotropical, Oriental, and Palearctic regions. Diagnostic drawings have been made for all species in the genus, and 27 species are known only from a single sex: 13 from females and 14 from males. To date, 47 species are known from Asia, including 29 from China. Among the Chinese *Phintella*, 16 are endemic and 12 are known from only a single sex: six from males and six from females (WSC 2020). Based on the continuous discovery of undescribed species in our recent fieldwork, it is likely that the true diversity of Chinese *Phintella* is much greater than currently known.

#### Phintella banna sp. nov.

Taxon classificationAnimaliaAraneaeSalticidae

334565A9-2F29-5D41-BAAD-CB8B32351FBE

http://zoobank.org/C3900F5F-68F0-48E1-805F-53069417C171

Figs 7 , 8

##### Type material.

***Holotype*** ♂ (IZCAS-Ar40608), China: Yunnan: Xishuangbanna, Mengla County, Menglun Town, Menglun Nature Reserve, Leprosy Village (21°53.59'N, 101°17.30'E, ca 550 m), 4.05.2019, Y. Tong et al. leg. ***Paratypes*** 5♀3♂ (IZCAS-Ar40609–40616), same data as holotype; 1♀3♂ (IZCAS-Ar40617–40620), Xishuangbanna Tropical Botanical Garden, tropical rainforest (21°55.35'N, 101°16.36'E, ca 610 m), 7.08.2018, C. Wang et al. leg.

##### Etymology.

The species name is derived from the name of the type locality; noun in apposition.

##### Diagnosis.

*Phintella banna* sp. nov. resembles *P. bifurcilinea* (Bösenberg & Strand, 1906) by the small size and the shape of the copulatory organs but can be distinguished by the following: 1) the lamellar process is visible in ventral view vs obscured in *P. bifurcilinea* (Żabka 1985: fig. 403); 2) the embolus is apically directed anteriorly in ventral view vs directed retrolaterally in *P. bifurcilinea* (Żabka 1985: fig. 403); 3) the chelicerae of the male are underdeveloped, the ratio of the length of the fang to the width of the paturon is about 1:1 vs well-developed chelicerae, with a ratio of almost 2.5:1 in *P. bifurcilinea* (Żabka 1985: fig. 406); 4) the copulatory openings are anterior to the spermathecae vs lateral to the spermathecae in *P. bifurcilinea* (Peng et al. 1993: fig. 527); 5) the copulatory ducts are approximately one-third of the spermathecal diameter vs less than one-fifth of the spermathecal diameter in *P. bifurcilinea* (Peng et al. 1993: fig. 527).

##### Description.

**Figure 7.**
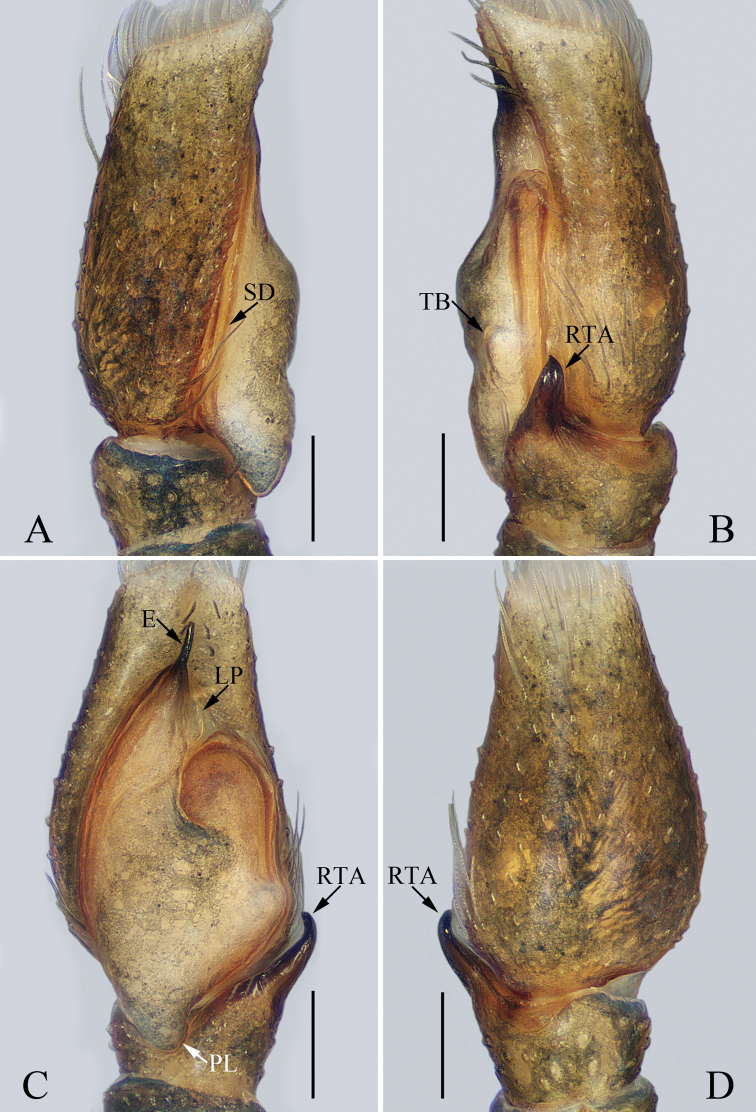
Male palp of the holotype of *Phintella banna* sp. nov. **A** prolateral **B** retrolateral **C** ventral **D** dorsal. Scale bars: 0.1.

**Figure 8.**
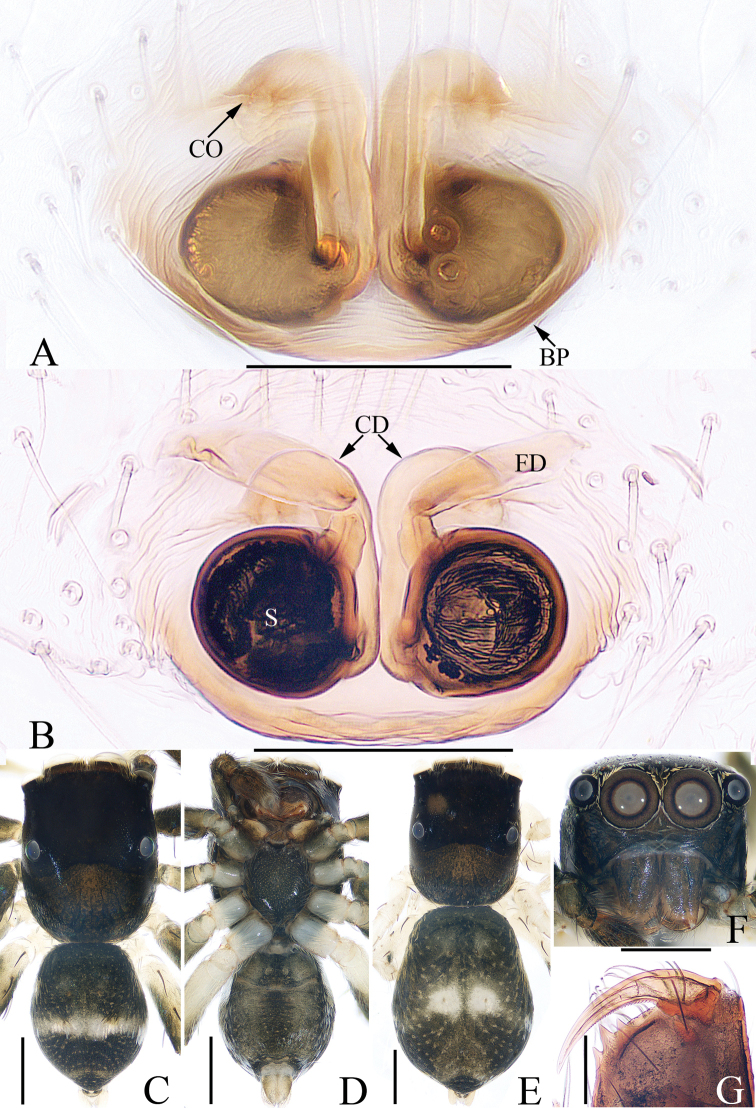
*Phintella banna* sp. nov., female paratype and male holotype **A** epigyne, ventral **B** vulva, dorsal **C** holotype habitus, dorsal **D** holotype habitus, ventral **E** paratype habitus, dorsal **F** holotype carapace, frontal **G** holotype chelicerae, dorsal. Scale bars: 0.1 (**A, B, G**); 0.5 (**C–F**).

**Male** (Figs 7, 8C, D, F, G). Total length 2.41. Carapace 1.34 long, 1.02 wide. Abdomen 1.12 long, 0.88 wide. Clypeus 0.10 high. Eye sizes and inter-distances: AME 0.33, ALE 0.17, PLE 0.15, AERW 1.01, PERW 0.98, EFL 0.64. Legs: I 2.73 (0.82, 0.98, 0.59, 0.34), II 2.66 (0.80, 0.93, 0.59, 0.34), III 3.25 (1.01, 1.07, 0.83, 0.34), IV 3.46 (1.10, 1.12, 0.90, 0.34). Carapace dark brown, slightly paler medially in the thoracic area, with yellow and pale scales on the cheeks. Clypeus dark brown. Fovea indistinct. Chelicerae yellow-brown to brown, with one retromarginal tooth and two promarginal teeth. Endites green-brown, pale yellow along the inner edge. Labium wider than long, paler terminally. Sternum green-brown, covered with thin setae. Legs pale to green-brown. Abdomen oval, dorsum hairy, with two pairs of muscle depressions and a transverse white band of scales in the center, followed by recurved dotted lines; venter colored as dorsum, darker medially.

***Palp*** (Fig. 7A–D): tibia wider than long, with a tapered RTA terminally curved towards the bulb and pointed apically in retrolateral view; bulb flat, with a lamellar process near the base of the embolus, approximately three times longer than wide, with a small subtriangular bump and a gently curved posterior lobe reaching the middle of the tibia in ventral view; sperm duct long, strongly curved anteriorly; embolus bar-shaped, almost equal to the length of the lamellar process.

**Female** (Fig. 8A, B, E). Total length 3.07. Carapace 1.29 long, 1.04 wide. Abdomen 1.76 long, 1.33 wide. Clypeus 0.10 high. Eye sizes and inter-distances: AME 0.32, ALE 0.18, PLE 0.16, AERW 1.04, PERW 1.01, EFL 0.67. Legs: I 2.31 (0.73, 0.80, 0.49, 0.29), II 2.25 (0.71, 0.76, 0.44, 0.34), III 2.88 (0.88, 0.93, 0.73, 0.34), IV 3.31 (1.02, 1.07, 0.88, 0.34). Habitus similar to that of the male except the transverse abdominal white band is indistinct laterally, and the dorsum of the abdomen with a brown, thin, longitudinal band in the middle.

***Epigyne*** (Fig. 8A, B): wider than long, with an arc-shaped basal plate; copulatory openings anteriorly located, slit-shaped, the distance between them almost equal to the spermathecal diameter; copulatory ducts curved anteriorly, and then descend along the longitudinal axis to connect to the postero-ento-lateral part of the spermathecae; spermathecae spherical, separated from each other by less than one-third their diameter; fertilization ducts well-developed, lamellar, anterior to spermathecae.

##### Distribution.

Known only from the type locality in Yunnan, China.

#### Phintella mii sp. nov.

Taxon classificationAnimaliaAraneaeSalticidae

B9B5EF5A-18F2-5D2F-B506-0F71B528DE03

http://zoobank.org/58C57CE9-D503-4CAF-BEE5-EEC57FF2D024

Figs 9 , 10

##### Type material.

***Holotype*** ♂ (IZCAS-Ar40621), China: Yunnan: Xishuangbanna, Mengla County, Menglun Town, Menglun Nature Reserve, Xishuangbanna Tropical Botanical Garden, tropical rainforest (21°55.35'N, 101°16.36'E, ca 610 m), 29.07.2018, X. Mi et al. leg. ***Paratype*** 1♀ (IZCAS-Ar40622), Palm Garden (21°55.47'N, 101°15.05'E, ca 550 m), 6.08.2018, C. Wang et al. leg.

##### Etymology.

The specific name is a patronym after Prof. Xiaoqi Mi (Tongren, China), one of the collectors of the new species; noun (name) in genitive case.

##### Diagnosis.

The male of *Phintella mii* sp. nov. resembles *P. aequipeiformis* Żabka, 1985 in the shape of the palp but can be distinguished by the following: 1) the distance between the embolus and cymbial tip is almost equal to the embolic length vs more than three times the embolic length in *P. aequipeiformis* (Luong et al. 2016: fig. 4A, D, E); 2) proximally, the RTA is strongly curved dorsally in retrolateral view vs straight in *P. aequipeiformis* (Luong et al. 2016: fig. 4B); 3) the chelicerae of the male are underdeveloped, the ratio of the length of the fang to the width of the paturon is about 1:1 vs well-developed chelicerae, with a ratio of almost 2.5:1 in *P. aequipeiformis* (Luong et al. 2016: fig. 3I). The female of this new species can be easily distinguished from other congeners by having an anterior subtriangular lamellar epigynal fold (Fig. 10A, F), which is absent in other species.

##### Description.

**Figure 9.**
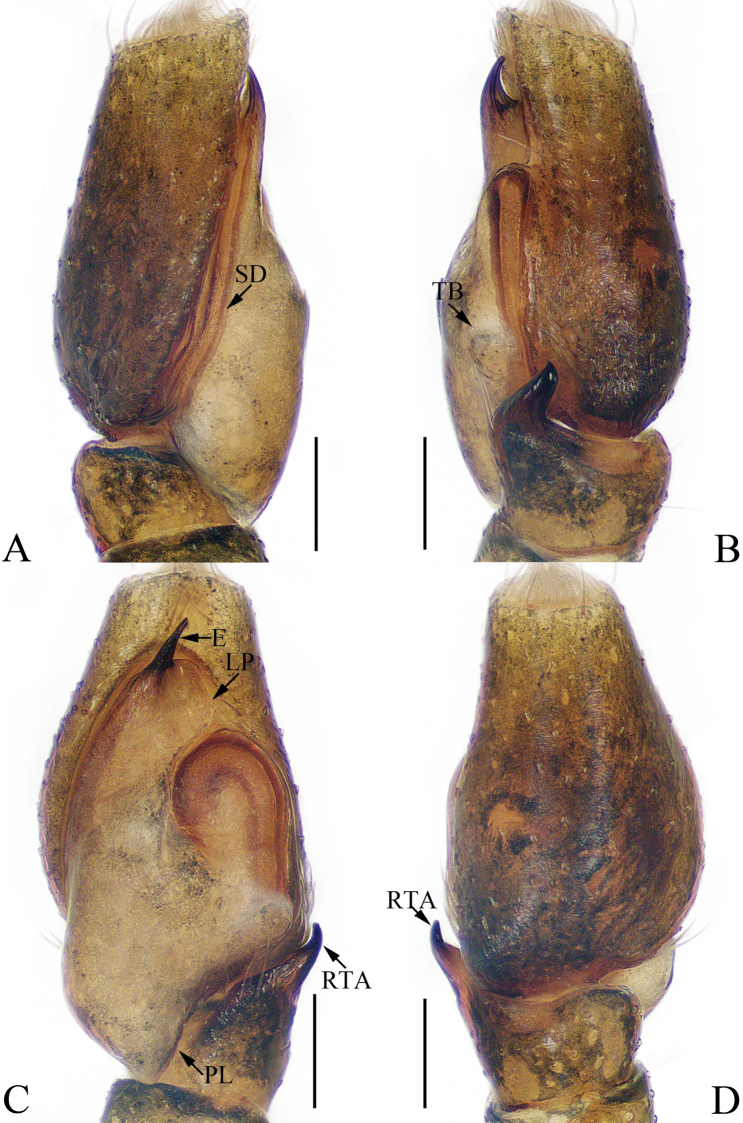
Male palp of the holotype of *Phintella mii* sp. nov. **A** prolateral **B** retrolateral **C** ventral **D** dorsal. Scale bars: 0.1.

**Figure 10.**
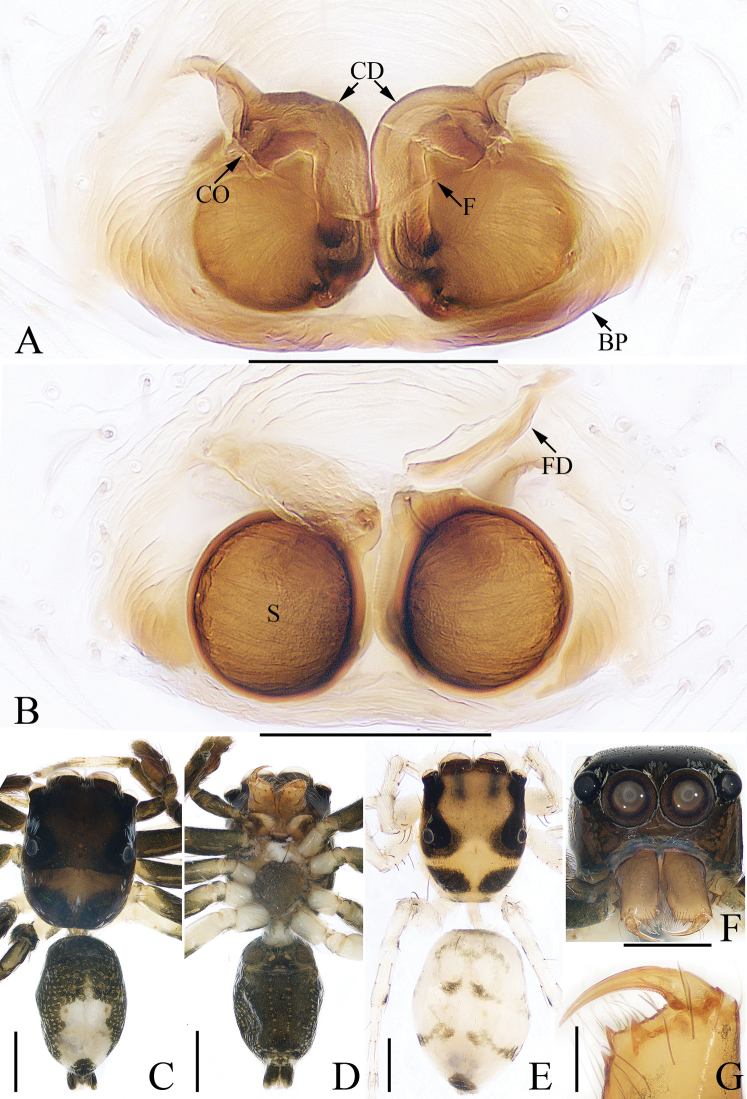
*Phintella mii* sp. nov., female paratype and male holotype **A** epigyne, ventral **B** vulva, dorsal **C** holotype habitus, dorsal **D** holotype habitus, ventral **E** paratype habitus, dorsal **F** holotype carapace, frontal **G** holotype chelicerae, dorsal. Scale bars: 0.1 (**A, B, G**); 0.5 (**C–F**).

**Male** (Figs 9, 10C, D, F, G). Total length 2.39. Carapace 1.05 long, 0.95 wide. Abdomen 1.15 long, 0.76 wide. Clypeus 0.05 high. Eye sizes and inter-distances: AME 0.30, ALE 0.15, PLE 0.14, AERW 0.93, PERW 0.91, EFL 0.59. Legs: I 2.46 (0.73, 0.90, 0.51, 0.32), II 2.20 (0.68, 0.76, 0.44, 0.32), III 2.68 (0.80, 0.88, 0.66, 0.34), IV 2.92 (0.90, 0.95, 0.73, 0.34). Carapace nearly square, bearing sparse white scales on cheeks, between both ALEs and PLEs and posteriorly, with a pair of indistinct stripes anteriorly and two pairs of round, dark markings near the PMEs and the posterior margin. Clypeus yellow-brown. Fovea longitudinal, short, bar-shaped. Chelicerae yellow, with one retromarginal tooth and two promarginal teeth. Endites yellow to green-brown. Labium dark brown. Sternum green-brown, heart-shaped, covered by dense, white setae. Legs pale to green-brown. Abdomen suboval, dorsum green-brown, speckled, with a nearly square white marking posteriorly, followed by a round, dark spot; venter dark brown, with a pair of longitudinal dotted lines medially.

***Palp*** (Fig. 9A–D): tibia wider than long, with a RTA proximally curved dorsally, and then curved towards bulb terminally, pointed at the tip in retrolateral view; bulb flat, with sperm duct strongly bent anteriorly; lamellar process small, nearly round, tegular bump located retrolatero-medially; embolus short, tapered, with a blunt tip directed antero-prolaterally in ventral view.

**Female** (Fig. 10A, B, E). Total length 3.04. Carapace 1.24 long, 1.01 wide. Abdomen 1.52 long, 1.12 wide. Clypeus 0.06 high. Eye sizes and inter-distances: AME 0.32, ALE 0.16, PLE 0.15, AERW 1.01, PERW 1.00, EFL 0.67. Legs: I 2.16 (0.68, 0.80, 0.44, 0.24), II 2.14 (0.68, 0.76, 0.46, 0.24), III missing, IV 3.05 (0.93, 1.05, 0.78, 0.29). Carapace yellow, with a pair of longitudinal dark stripes anteriorly in eye field and two pairs of dark brown patches close to PMEs and the posterior margin of the thoracic area. Abdomen suboval, dorsum pale, with indistinct transverse brown stripes medially and a round, brown spot at the terminus; venter colored as dorsum.

***Epigyne*** (Fig. 10A, B): with a slightly recurved basal plate; copulatory openings located anteriorly, obscured by an inverted subtriangular lamellar epigynal fold; copulatory ducts extending transversely before extending posteriorly along the longitudinal axis, and then curving to connected to the dorsoectal surface of spermathecae; spermathecae almost round, separated from each other by less than one-third their diameter; fertilization ducts well-developed, located anteriorly on spermathecae.

##### Distribution.

Known only from the type locality in Yunnan, China.

##### Comments.

The female is paired with the holotype because it shares similar carapace markings with the male. These markings differ from other congeners known only from a single male from Xishuangbanna. However, the male and female have different abdominal markings, so the pairing requires further confirmation.

#### Genus Simaetha

Taxon classificationAnimaliaAraneaeSalticidae

Strand, 1932

1525B38F-DD6E-5A52-88BB-FF15A131CF0A

##### Type species.

*Simaetha thoracica* Thorell, 1881

##### Comments.

*Simaetha* is represented by 19 nominal species distributed in East and Southeast Asia, New Guinea, and Australia (WSC 2020). All species are endemic except *S. knowlesi* Żabka, 1994, *S. paetula* (Keyserling, 1882), *S. robustior* (Keyserling, 1882), *S. tenuidens* (Keyserling, 1882), and *S. tenuior* (Keyserling, 1882), known from both New Guinea and Australia. Although a comprehensive work was done by Żabka (1994), the genus is rather poorly studied, with 11 of its species known only from a single sex – five from females and six from males – and four species lack diagnostic drawings. To date, only the endemic species, *S. gongi* Peng, Gong & Kim, 2000, has been recorded from China.

#### Simaetha menglun sp. nov.

Taxon classificationAnimaliaAraneaeSalticidae

D5902F0C-9CA6-5774-95B2-902375A0D815

http://zoobank.org/594A88B2-52FD-41BA-B066-97AB35F12359

Figs 11 , 12

##### Type material.

***Holotype*** ♂ (IZCAS-Ar40623), China: Yunnan: Xishuangbanna, Mengla County, Menglun Town, Menglun Nature Reserve, Leprosy Village (21°53.62'N, 101°18.25'E, ca 520 m), 29.04.2019, Y. Tong et al. leg. ***Paratype*** 1♀ (IZCAS-Ar40624), same data as holotype.

##### Etymology.

The species name is derived from the name of the type locality; noun in apposition.

##### Diagnosis.

*Simaetha menglun* sp. nov. resembles *S. pengi* sp. nov. in the shape of the copulatory organs but can be easily distinguished by the shape of the habitus, the color pattern, and the cheliceral dentition. *S. menglun* sp. nov. resembles *S. gongi* Peng, Gong & Kim, 2000 by the general shape of the copulatory organs and the short palpal tibia but can be distinguished by the following: 1) the tip of the embolus is curved in ventral view vs straight in *S. gongi* (Peng et al. 2000: fig. 14); 2) the epigynal hood is about one and a half times longer than wide vs almost two times wider than long in *S. gongi* (Peng et al. 2000: fig. 11); 3) the chelicerae of the female have one bifurcated retromarginal tooth vs two retromarginal teeth in *S. gongi* (Peng et al. 2000: fig. 10). The male of *S. menglun* sp. nov. also somewhat resembles *S. deelemanae* Zhang, Song & Li, 2003 in the shape of the palp, but can be easily distinguished by the palpal tibia which is wider than long rather than distinctly longer than wide in *S. deelemanae* (Zhang et al. 2003: fig. 7C, D).

##### Description.

**Figure 11.**
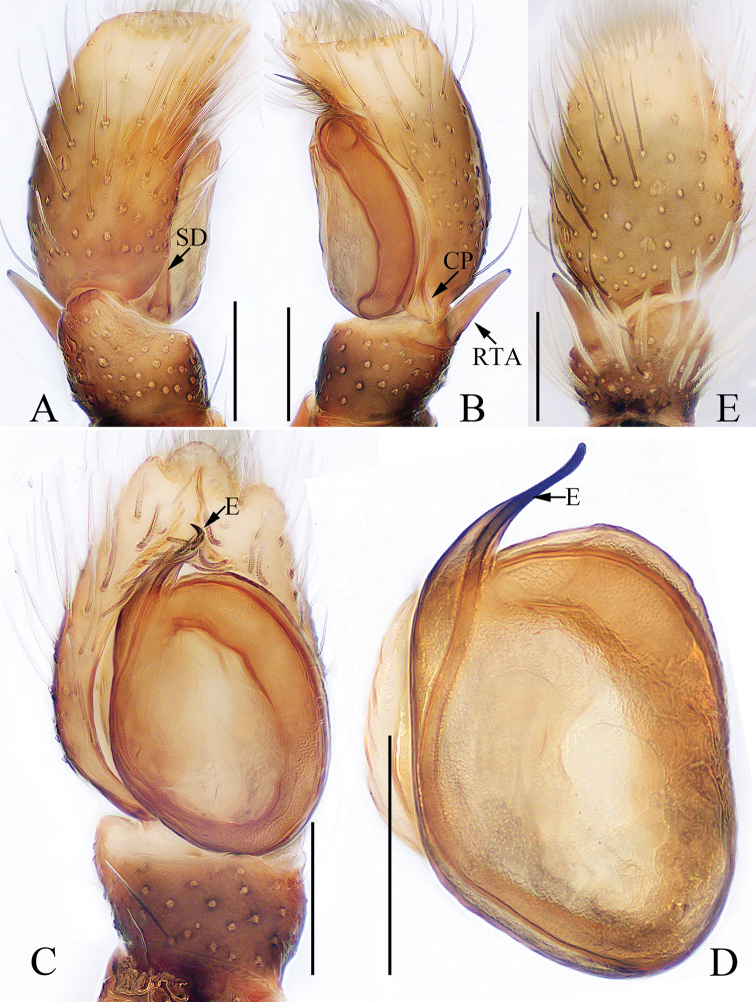
Male palp of the holotype of *Simaetha menglun* sp. nov. **A** prolateral **B** retrolateral **C** ventral **D** bulb, ventral **E** dorsal. Scale bars: 0.1.

**Figure 12.**
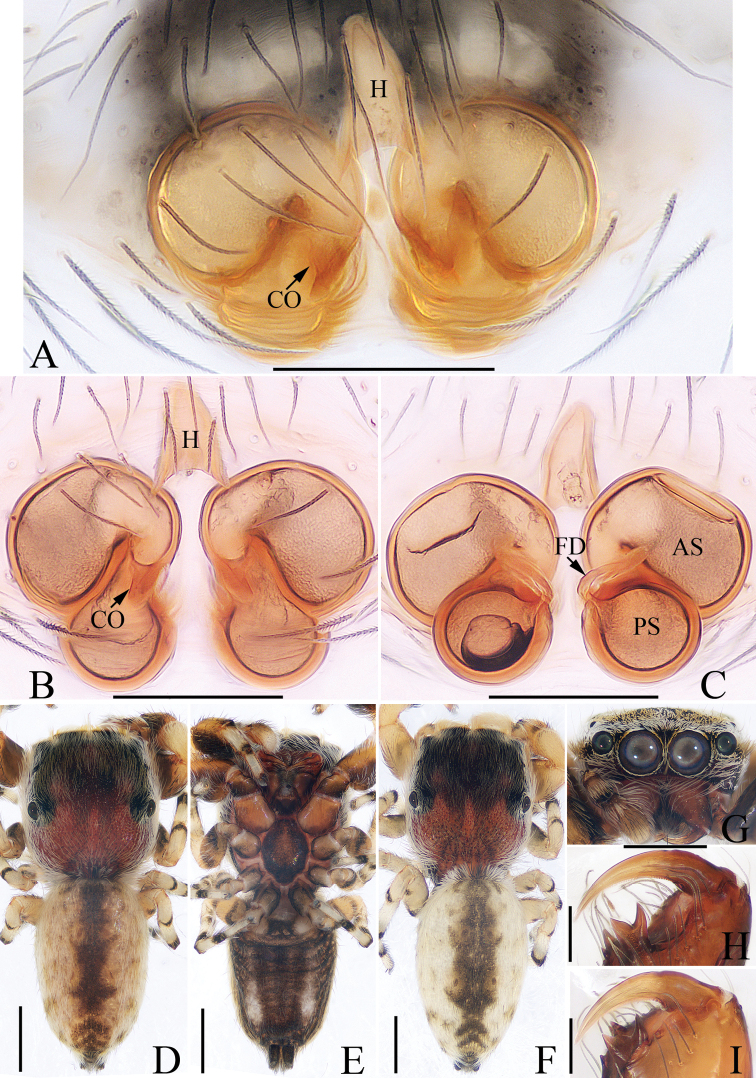
*Simaetha menglun* sp. nov., female paratype and male holotype **A, B** epigyne, ventral **C** vulva, dorsal **D** holotype habitus, dorsal **E** holotype habitus, ventral **F** paratype habitus, dorsal **G** holotype carapace, frontal **H** holotype chelicerae, dorsal **I** paratype chelicerae, dorsal. Scale bars: 0.1 (**A–C, H–I**); 0.5 (**D–G**).

**Male** (Figs 11, 12D, E, G, H). Total length 2.49. Carapace 1.07 long, 1.01 wide. Abdomen 1.24 long, 0.88 wide. Clypeus 0.01 high. Eye sizes and inter-distances: AME 0.26, ALE 0.13, PLE 0.13, AERW 0.89, PERW 0.98, EFL 0.61. Legs: I 1.95 (0.68, 0.76, 0.29, 0.22), II 1.49 (0.49, 0.54, 0.24, 0.22), III 1.34 (0.44, 0.44, 0.24, 0.22), IV 1.79 (0.59, 0.61, 0.32, 0.27). Carapace nearly square, red-brown, with an irregular dark patch in the middle of the cephalic part, bearing dense, white setae on the cheeks, and dense, white and yellow setae dorsally. Clypeus dark. Fovea indistinct. Chelicerae red-brown, with two promarginal teeth and one retromarginal tooth bifurcated into two pointed tips. Endites and labium dark brown. Sternum colored as labium, covered with thin setae. Legs yellow to yellow-brown, with dark rings; legs I with dark, inflated tibia bearing dense setae ventrally. Abdomen elongated, dorsum with longitudinal, irregular, dark-brown stripe extending posteriorly from the anterior margin, followed by two chevrons, covered by dense, thin, white setae laterally; venter dark brown, with a pair of longitudinal white bands laterally.

***Palp*** (Fig. 11A–E): tibia wider than long, with white scales dorsally and a lamellar RTA that tapers to a pointed tip which is almost directed towards 1:30 o’clock in retrolateral view; cymbium hirsute, with a proximo-retrolateral triangular process near the RTA base; bulb round and flat; embolus originates from a plate-like base, superimposed on the surface of the bulb, apically curved towards the prolateral side.

**Female** (Fig. 12A–C, F, I). Total length 3.01. Carapace 1.28 long, 1.09 wide. Abdomen 1.66 long, 1.23 wide. Clypeus 0.01 high. Eye sizes and inter-distances: AME 0.29, ALE 0.14, PLE 0.14, AERW 0.97, PERW 1.09, EFL 0.69. Legs: I 1.95 (0.66, 0.76, 0.29, 0.24), II 1.63 (0.51, 0.61, 0.27, 0.24), III 1.53 (0.49, 0.51, 0.29, 0.24), IV 2.03 (0.66, 0.76, 0.34, 0.27). Habitus similar to that of male except paler, and the chelicerae have three promarginal teeth.

***Epigyne*** (Fig. 12A–C): wider than long, with an anterior hood approximately 1.5 times longer than wide; copulatory openings slit-like, located medially; copulatory ducts short, connected to the anterior chambers of the spermathecae; spermathecae divided into two round chambers; fertilization ducts connected to the anterior part of the posterior chambers of the spermathecae, extending obliquely.

##### Distribution.

Known only from the type locality in Yunnan, China.

##### Comments.

This species and *Simaetha pengi* sp. nov. are placed into this genus due to sharing a similar habitus and similar copulatory organs with *S. gongi* and *S. deelemanae* which occur in China and Singapore.

#### Simaetha pengi sp. nov.

Taxon classificationAnimaliaAraneaeSalticidae

EE2000AE-677F-52FB-B205-866C9BFEB5FF

http://zoobank.org/DFE073DC-F938-4BEF-8492-B35D8716F8BA

Figs 13 , 14

##### Type material.

***Holotype*** ♂ (IZCAS-Ar40625), China: Yunnan: Xishuangbanna, Mengla County, Menglun Town, Menglun Nature Reserve, Leprosy Village (21°53.62'N, 101°18.25'E, ca 520 m), 29.04.2019, Y. Tong et al. leg. ***Paratypes*** 2♀ (IZCAS-Ar40626–40627), same data as holotype.

##### Etymology.

The specific name is a patronym in honor of Prof. Xianjin Peng (Changsha, China), who has produced many important taxonomic works on Chinese jumping spiders; noun (name) in genitive case.

##### Diagnosis.

*Simaetha pengi* sp. nov. resembles *S. gongi* Peng, Gong & Kim, 2000 but can be distinguished by the apically curved embolus (vs straight), the longer than wide epigynal hood (vs wide than long), and the bifurcated retromarginal tooth of the female chelicerae (vs not bifurcated). The male of *S. menglun* sp. nov. also somewhat resembles *S. deelemanae* Zhang, Song & Li, 2003 in the shape of the palp but can be easily distinguished by the short palpal tibia, which is wider than long, but distinctly longer than wide in *S. deelemanae*.

##### Description.

**Figure 13.**
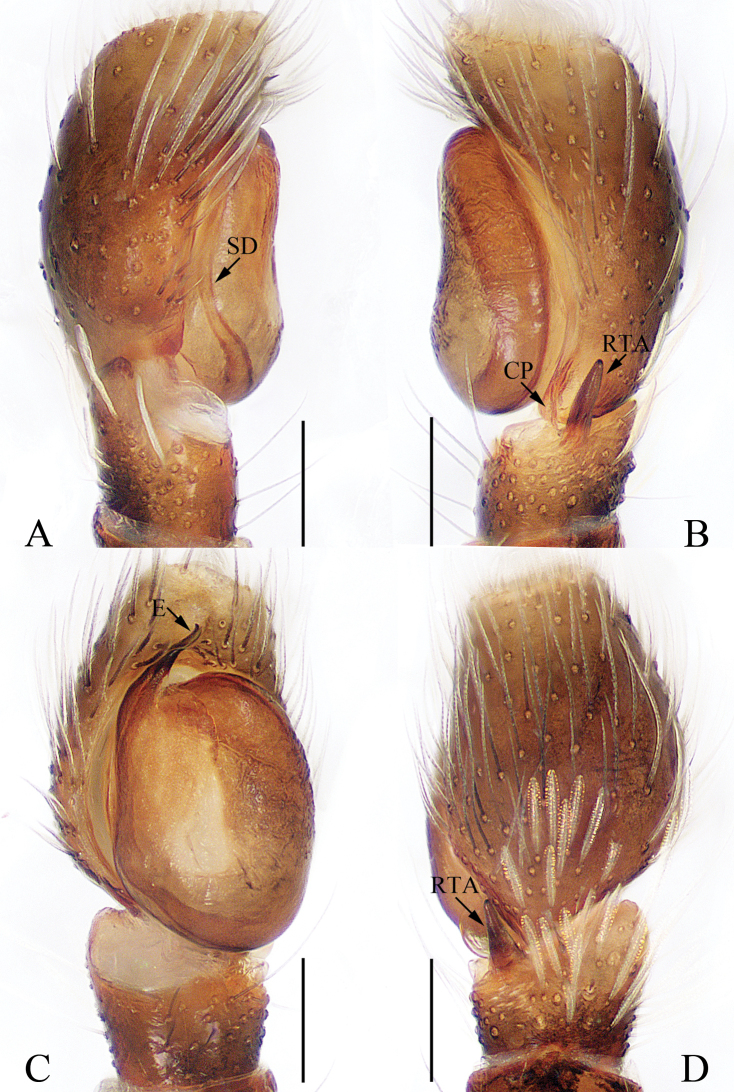
Male palp of the holotype of *Simaetha pengi* sp. nov. **A** prolateral **B** retrolateral **C** ventral **D** dorsal. Scale bars: 0.1.

**Figure 14.**
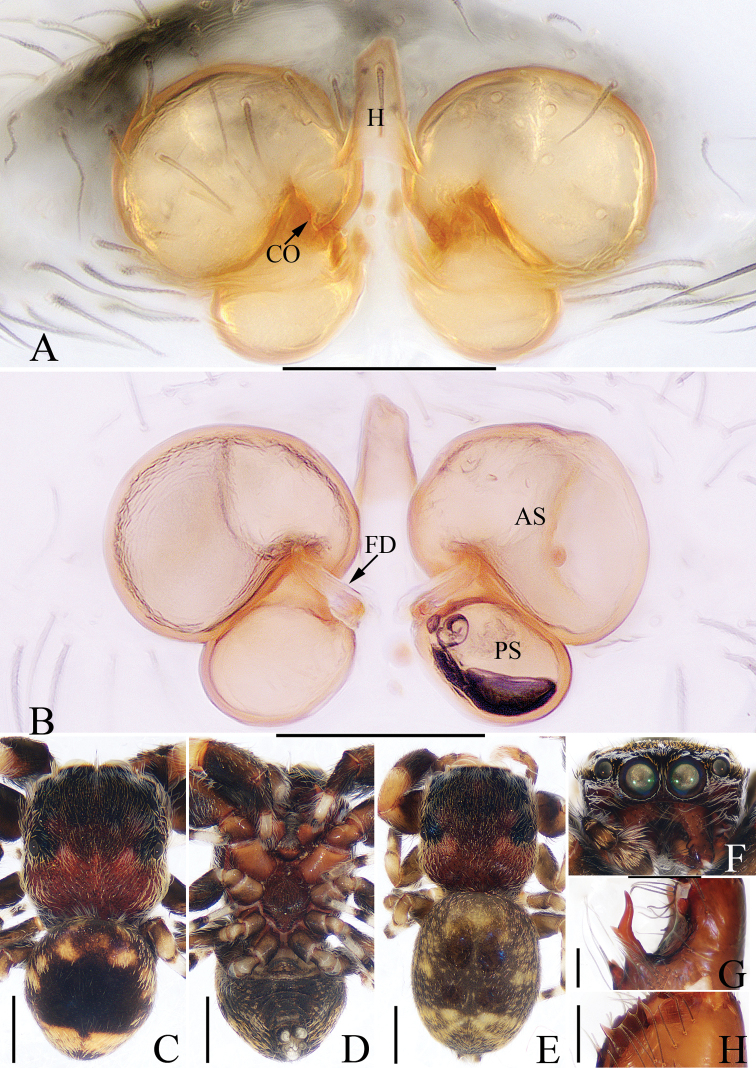
*Simaetha pengi* sp. nov., female paratype and male holotype **A** epigyne, ventral **B** vulva, dorsal **C** holotype habitus, dorsal **D** holotype habitus, ventral **E** paratype habitus, dorsal **F** holotype carapace, frontal **G** holotype chelicerae, dorsal **H** paratype chelicerae, dorsal. Scale bars: 0.1 (**A, B, G, H**); 0.5 (**C–F**).

**Male** (Figs 13, 14C, D, F, G). Total length 2.17. Carapace 1.15 long, 1.11 wide. Abdomen 1.11 long, 1.01 wide. Clypeus 0.02 high. Eye sizes and inter-distances: AME 0.28, ALE 0.14, PLE 0.13, AERW 0.93, PERW 0.97, EFL 0.62. Legs: I 2.39 (0.78, 1.00, 0.34, 0.27), II 1.80 (0.56, 0.63, 0.34, 0.27), III 1.66 (0.51, 0.54, 0.34, 0.27), IV 2.00 (0.68, 0.71, 0.32, 0.29). Carapace almost square, red-brown, with a pair of indistinct dark brown patches medially on cephalic part, bearing dense, white setae forming two parallel transverse stripes on cheeks and golden-brown setae, two clusters of white setae near the PMEs on the dorsum. Clypeus dark brown. Fovea indistinct. Chelicerae red-brown, with two promarginal teeth and one retromarginal, pillar-shaped tooth bifurcated distally, with additional digitiform and round protuberances at the promargin and retromargin, respectively. Endites and labium colored as chelicerae. Sternum darker than endites, covered with pale, thin setae. Legs I with inflated, dark femora and tibiae, the latter bearing dense ventral setae, patellae yellow, tarsus pale yellow; other legs yellow with dark rings, except femora dark brown. Abdomen oval, dorsum dark medially, with spots of yellow scales anterolaterally and a yellow longitudinal band of scales posteriorly; venter brown.

***Palp*** (Fig. 13A–D): tibia almost as long as wide, with transparent white scales dorsally, and a RTA less than tibial length, tapered to a slightly pointed tip, directed towards 1:00 o’clock in retrolateral view; cymbium hirsute, with a proximo-retrolateral triangular process and sparse, transparent white scales proximo-dorsally; bulb flat, with sperm duct extending along the margin; embolus arises from a plate-like base, superimposed on the surface of the bulb, slightly curved apically.

**Female** (Fig. 14A, B, E, H). Total length 2.48. Carapace 1.10 long, 1.03 wide. Abdomen 1.40 long, 1.13 wide. Clypeus 0.02 high. Eye sizes and inter-distances: AME 0.26, ALE 0.12, PLE 0.12, AERW 0.86, PERW 1.01, EFL 0.59. Legs: I 1.98 (0.71, 0.76, 0.27, 0.24), II 1.64 (0.54, 0.59, 0.27, 0.24), III 1.53 (0.49, 0.51, 0.29, 0.24), IV 2.03 (0.71, 0.73, 0.32, 0.27). Habitus similar to that of male, except paler, and the chelicerae have one promarginal tooth and one retromarginal fissident with three cusps.

***Epigyne*** (Fig. 14A, B): wider than long, with an anterior bell-shaped hood approximately 1.5 times longer than wide; copulatory openings slit-like, located medially, separated from each other by approximately 1.5 times the width of the hood; copulatory ducts short; spermathecae divided into two round chambers; fertilization ducts connected to the anterior part of the posterior chambers of the spermathecae.

##### Distribution.

Known only from the type locality in Yunnan, China.

## Supplementary Material

XML Treatment for Charippus

XML Treatment for Charippus yinae

XML Treatment for Chinattus

XML Treatment for Chinattus inflatus

XML Treatment for Indomarengo

XML Treatment for Indomarengo yui

XML Treatment for Phintella

XML Treatment for Phintella banna

XML Treatment for Phintella mii

XML Treatment for Simaetha

XML Treatment for Simaetha menglun

XML Treatment for Simaetha pengi
